# Histone de-acetylase inhibitors: a promising future for cancer treatment?

**DOI:** 10.1186/1750-9378-8-10

**Published:** 2013-03-11

**Authors:** Raja Samir Khan, Harris Hameed, Ramsha Ali Bhutta, Abdul Nafey Kazi, Haris Riaz

**Affiliations:** 1 Aga Khan University, Karachi, Pakistan; 2 Glasgow University, Glasgow, Scotland; 3 Dow Medical College, Karachi, Pakistan; 4 Department of Medicine, Civil Hospital Karachi, Karachi, Pakistan

## 

Histone de-acetylase proteins or lysine de-acetylases, represent a group of enzymes regulating DNA and gene expression by eliminating acetyl groups (de-acetylation) from lysine amino acids on histones and non-histone proteins [1,2]. Histone de-acetylase functions normally by being involved in a series of cellular pathways like cell growth, cell cycle, signal transduction, notch signaling pathway and especially transcription [3]. Although these functions allows histone de-acetylases to control expression of proteins involved in cancer initiation and cancer progression, abnormal acetylation of histone tails may occur with the resulting transcriptional lesions disrupting the apoptotic program of cells and leading to neoplasia [4,5]. To counter, development of antineoplastic agents called histone de-acetylase inhibitors (HDIs) have been introduced to interact with the catalytic site and blocking substrate access of histone de-acetylases in proliferation of tumor cells [6]. This anti-proliferative effect of HDIs in down regulation of BMI1 and c-MYC protein levels has shown promising results in treatment of the incurable AML as well as silencing estrogen receptor alpha in prevention of breast cancer [7,8].

The precise molecular actions of HDIs are unclear with epigenetic pathways the proposed mechanism [9].

HDIs mainly induce activation of both intrinsic and extrinsic apoptotic pathways of neoplastic cells by affecting protein stability, protein-protein interactions through interference with the function of cell cycle proteins such as p21, inhibition of signaling pathways implicated with Raf/MEK and activation of Reactive Oxygen Species [10,11]. In stress, HDIs acetylate DNA damage-response proteins, such as Ku70, causing the translocation of BAX to the mitochondria and activating apoptosis [12]. Recently they have been known to cause autophagy of signaling pathways like mTOR, AIF which is a major development [13]. Some studies have also shown HDIs contributing to growth suppression of primary tumors by enhancing tumor-cell’s immunogenicity via transcriptional activation of MHC (1/2) proteins and suppression of tumor angiogenesis by inhibition of hypoxia-induced VEGF expression [14,15].

Despite the intrinsic anticancer potential, there are noteworthy limitations of these promising antineoplastic agents [11]. Combinations of HDIs with other cancer modalities such as anthracyclines have been found to be effective in pre-clinical and clinical evaluation; however, their additive effect has also led to potential complexities such as resistance to anthracycline (doxorubicin) in leukaemia cells and augmentation of cardiac toxicity [16]. Through modulation, HDIs also reactivate some latent viruses like human herpesvirus-6 predisposing to reinfection [17]. However, resistance to HDACi-induced transformed cell death, as observed in clinical trials of human bladder carcinoma cells and prostate cancer cells and non-specific action against a large group of Histone de-acetylases forms the major limitations of these agents [15]. Basis of this resistance is yet to be understood [15].

Regardless of the Food and Drug Administration (FDA) approval of Vorinostat (SAHA) and Romidepsin (ISTODAX) for the promising treatment of cutaneous T cell lymphoma, usage of HDIs as mono-therapies in other types of cancer has had moderate effects [18]. Preclinical studies on cancers have concluded better synergistic and additive effects of HDIs with combination of chemotherapeutic drugs by helping improve HDIs therapeutic index [18]. With complete and proper understanding of the major limitations of HDIs, their antitumor efficacy can be achieved enhancing the novel use of these anticancer drugs for the future treatments just like their current use as mood stabilizers and anti-epileptics.

## Competing interests

The authors declare that they have no competing interests.

## Authors’ contributions

RSK: wrote article. HH: wrote article. RAB: made changes, proof read. ANK: idea and search, made revision changes. HR: made revision changes. All authors read and approved the final manuscript.

## References

[B1] Bottomley MJ Lo Surdo P Di Giovine P Cirillo A Scarpelli R Ferrigno F Jones P Neddermann P Structural and functional analysis of the human HDAC4 catalytic domain reveals a regulatory structural zinc-binding domain J Biol Chem 2008 283 39 26694 26704 10.1074/jbc.M803514200 18614528 PMC3258910

[B2] Choudhary C Lysine acetylation targets protein complexes and co-regulates major cellular functions Science 2009 325 5942 834 840 10.1126/science.1175371 19608861

[B3] Bertos NR Wang AH Yang XJ Class II histone deacetylases: structure, function, and regulation Biochem Cell Biol 2001 79 3 243 252 10.1139/o01-032 11467738

[B4] Glozak MA Seto E Histone deacetylases and cancer Oncogene 2007 26 5420 5432 10.1038/sj.onc.1210610 17694083

[B5] Jacobson S Pillus L Modifying chromatin and concepts of cancer Curr Opin Genet Dev 1999 9 2 175 184 10.1016/S0959-437X(99)80027-6 10322138

[B6] Johnstone R Histone-deacetylase inhibitors: novel drugs for the treatment of cancer Nat Rev Drug Discov 2002 1 4 287 299 10.1038/nrd772 12120280

[B7] Romanski A Schwarz K Keller M Wietbrauk S Vogel A Roos J Oancea C Brill B Krämer OH Serve H Ruthardt M Bug G Deacetylase inhibitors modulate proliferation and self-renewal properties of leukemic stem and progenitor cells Cell Cycle 2012 11 17 3219 26 10.4161/cc.21565 22895185 PMC3466521

[B8] Zhang Z Yamashita H Toyama T Quantitation of HDAC1 mRNA expression in invasive carcinoma of the breast Breast Cancer Res 2005 94 1 11 16 10.1007/s10549-005-6001-1 16172792

[B9] Monneret C Histone deacetylase inhibitors for epigenetic therapy of cancer Anticancer Drugs 2007 18 4 363 370 10.1097/CAD.0b013e328012a5db 17351388

[B10] Drummond DC Noble CO Kirpotin DB Guo Z Scott GK Benz CC Clinical development of histone deacetylase inhibitors as anticancer agents Annu Rev Pharmacol Toxicol 2005 45 495 528 10.1146/annurev.pharmtox.45.120403.095825 15822187

[B11] Minucci S Pelicci PG Histone deacetylase inhibitors and the promise of epigenetic (and more) treatments for cancer Nature 2006 6 38 51 10.1038/nrc1779 16397526

[B12] Rosato RR Grant S Histone Deactetylase inhibitors in cancer therapy Cancer Biol Ther 2009 2 1 30 37 12673114 10.4161/cbt.190

[B13] Robert T HDACs link the DNA damage response, processing of double-strand breaks and autophagy Nature 471 74 79 21368826 10.1038/nature09803 PMC3935290

[B14] Magner WJ Activation of MHC class I, II, and CD40 gene expression by histone deacetylase inhibitors J Immunol 2000 165 12 7017 7024 11120829 10.4049/jimmunol.165.12.7017

[B15] Xu WS Parmigiani RB Marks PA Histone deacetylase inhibitors: molecular mechanisms of action Oncogene 2007 26 5541 5552 10.1038/sj.onc.1210620 17694093

[B16] Ververis K Karagiannis T *Overview of the classical histone deacetylase enzymes and histone deacetylase inhibitors* ISRN Cell Biology 2012 2012 12

[B17] Arbuckle JH Medveczky PG The molecular biology of human herpesvirus-6 latency and telomere integration Microbes Infect 2011 13 8–9 731 741 21458587 10.1016/j.micinf.2011.03.006 PMC3130849

[B18] Miller CP Singh MM Rivera-Del Valle N Manton CA Chandra J Therapeutic strategies to enhance the anticancer efficacy of histone deacetylase inhibitors J Biomed Biotechnol 2011 2011 514261 21765634 10.1155/2011/514261 PMC3134392

